# FGF Signaling Pathway in the Developing Chick Lung: Expression and Inhibition Studies

**DOI:** 10.1371/journal.pone.0017660

**Published:** 2011-03-11

**Authors:** Rute S. Moura, José P. Coutinho-Borges, Ana P. Pacheco, Paulo O. daMota, Jorge Correia-Pinto

**Affiliations:** School of Health Sciences, Life and Health Sciences Research Institute (ICVS), University of Minho, Braga, Portugal; Texas A&M University, United States of America

## Abstract

**Background:**

Fibroblast growth factors (FGF) are essential key players during embryonic development. Through their specific cognate receptors (FGFR) they activate intracellular cascades, finely regulated by modulators such as Sprouty. Several FGF ligands (FGF1, 2, 7, 9, 10 and 18) signaling through the four known FGFRs, have been implicated in lung morphogenesis. Although much is known about mammalian lung, so far, the avian model has not been explored for lung studies.

**Methodology/Principal Findings:**

In this study we provide the first description of *fgf10*, *fgfr1-4* and *spry2* expression patterns in early stages of chick lung development by *in situ* hybridization and observe that they are expressed similarly to their mammalian counterparts. Furthermore, aiming to determine a role for FGF signaling in chick lung development, *in vitro* FGFR inhibition studies were performed. Lung explants treated with an FGF receptor antagonist (SU5402) presented an impairment of secondary branch formation after 48 h of culture; moreover, abnormal lung growth with a cystic appearance of secondary bronchi and reduction of the mesenchymal tissue was observed. Branching and morphometric analysis of lung explants confirmed that FGFR inhibition impaired branching morphogenesis and induced a significant reduction of the mesenchyme.

**Conclusions/Significance:**

This work demonstrates that FGFRs are essential for the epithelial-mesenchymal interactions that determine epithelial branching and mesenchymal growth and validate the avian embryo as a good model for pulmonary studies, namely to explore the FGF pathway as a therapeutic target.

## Introduction

Fibroblast growth factors (FGF) are secreted proteins that play an important role in multiple biological processes, such as proliferation, survival, migration and differentiation, as well as in the morphogenesis of branching organs such as the lung, kidney and pancreas, among others [1], [2]. FGF signaling depends on the activation of specific cell surface receptors (FGFR) encoded by four distinct genes, *fgfr1-4*. Due to alternative splicing, these can produce numerous FGFR isoforms that differ in their binding affinity towards the FGF ligands. FGFRs are single-pass transmembrane proteins with tyrosine kinase activity (RTK) which, upon ligand binding to the extracellular domain of the receptor, initiate a signal transduction cascade (Ras-MAP kinase, PI3 kinase/Akt) that ultimately results in gene expression modification [3], [4]. *fgf* and *fgfr* expression depend on cell type and physiological conditions and are strictly regulated in both time and space. In fact, when mutated or misexpressed, the modified genes can cause developmental disorders and can also lead to cancer [5], [6]. The FGF-FGFR interaction requires the intervention of heparin or heparin sulfate proteoglycans in order to stabilize the formation of a receptor dimer bound to the FGF molecules. The FGF-FGFR pathway is precisely controlled by modulators of RTK signaling, including members of the Sprouty (Spry) family of proteins that inhibit FGF-induced MAPK activation to varying degrees. Moreover, Spry modulation of RTK signaling is very flexible and highly cell- and context-dependent, and its expression is induced by the growth factor cascades that it regulates [7].

In the embryonic lung, FGF signaling is an essential component of the regulatory networks operating between epithelium and mesenchyme at several stages of development and its perturbation results in dramatic abnormalities of epithelial branching and differentiation. Only a restricted number of FGF ligands are present in the embryonic lung (FGF1, 2, 7, 9, 10 and 18; reviewed in [8]), and only FGF10 has been shown to be absolutely essential for lung formation, as *fgf10* knockout mice fail to develop lungs, leading to neonatal death [9]. During morphogenesis, *fgf10* is dynamically expressed in the lung mesenchyme surrounding the distal buds, and acts as a chemoattractant of lung epithelium controlling induction and directional outgrowth through its specific cell surface receptor (FGFR2b) [10], [11]. Targeted deletion of FGFR2b also prevents branching, causing the trachea to terminate as a blind sac [12]. The same occurs in *fgf10* knockouts, meaning that the fully functional pathway is necessary for lung formation. Furthermore, FGF signaling in the lung is regulated by Spry2, a RTK modulator that inhibits lung growth and morphogenesis [13]. Abrogation of *spry2* expression stimulates lung branching morphogenesis and increases epithelial proliferation [14]. Conversely, *spry2* overexpression results in reduced branching and decreased epithelial cell proliferation [13].

In the chicken embryo, *Gallus gallus* variant *domesticus*, lung buds become evident on day 3.5 of embryogenesis, appearing as paired protuberances on the lateroventral aspect of the foregut of the developing embryo [15]. Endodermal bud formation and septation of the respiratory tract and esophagus is defined by Tbx4 expression, which is present in the visceral mesoderm of the lung primordium, by activation of mesodermal Fgf10 expression. This signaling system is of great importance in the events that lead to primary bud formation [16]. Later, the single bud divides into left and right primordial lungs that progressively elongate caudally while separating and shifting towards the coelomic cavity [15]. As development proceeds, branching morphogenesis occurs along with the formation of the air sacs, in the ventral region. The developing respiratory tract presents region-specific mesenchymal expression of the *Hoxb* genes, *Bmp-2*, *Bmp-4*, *Wnt-5a*, and *Wnt-11* [17], that govern differences between dorsal and ventral compartments of the lung. Furthermore, variations in the rate of diffusion of FGF10 between ventral and dorsal regions, due to differential levels of heparin sulfate proteoglycans and a special mesenchymal structure, seem to account for ventral cyst formation and dorsal branching morphogenesis [18].

In this report, we have examined transcript location of *fgf10*, *fgfr1*, *fgfr2*, *fgfr3*, *fgfr4* and *spry2* by *in situ* hybridization at early stages of chick lung development. FGFR *in vitro* inhibition studies were also performed and branching and morphometric analysis were carried out in order to assess the role of FGF signaling pathway in chick lung development.

## Results

### Expression pattern of *fgf10* during chick lung development

In order to analyze the expression of some members of the FGF signaling pathway in early stages of chick lung development, whole mount *in situ* hybridization (ISH) experiments were performed, after which representative examples of hybridized lungs from different stages of development were sectioned for histology. Embryos with 4 to 6 days of incubation were collected and lungs were carefully dissected and staged according to the number of secondary bronchi formed: b1 stage corresponds to lungs with only one secondary bronchus, at b2 stage the lung presents two secondary bronchi and at b3 stage it presents three secondary bronchi.

**Figure 1:**
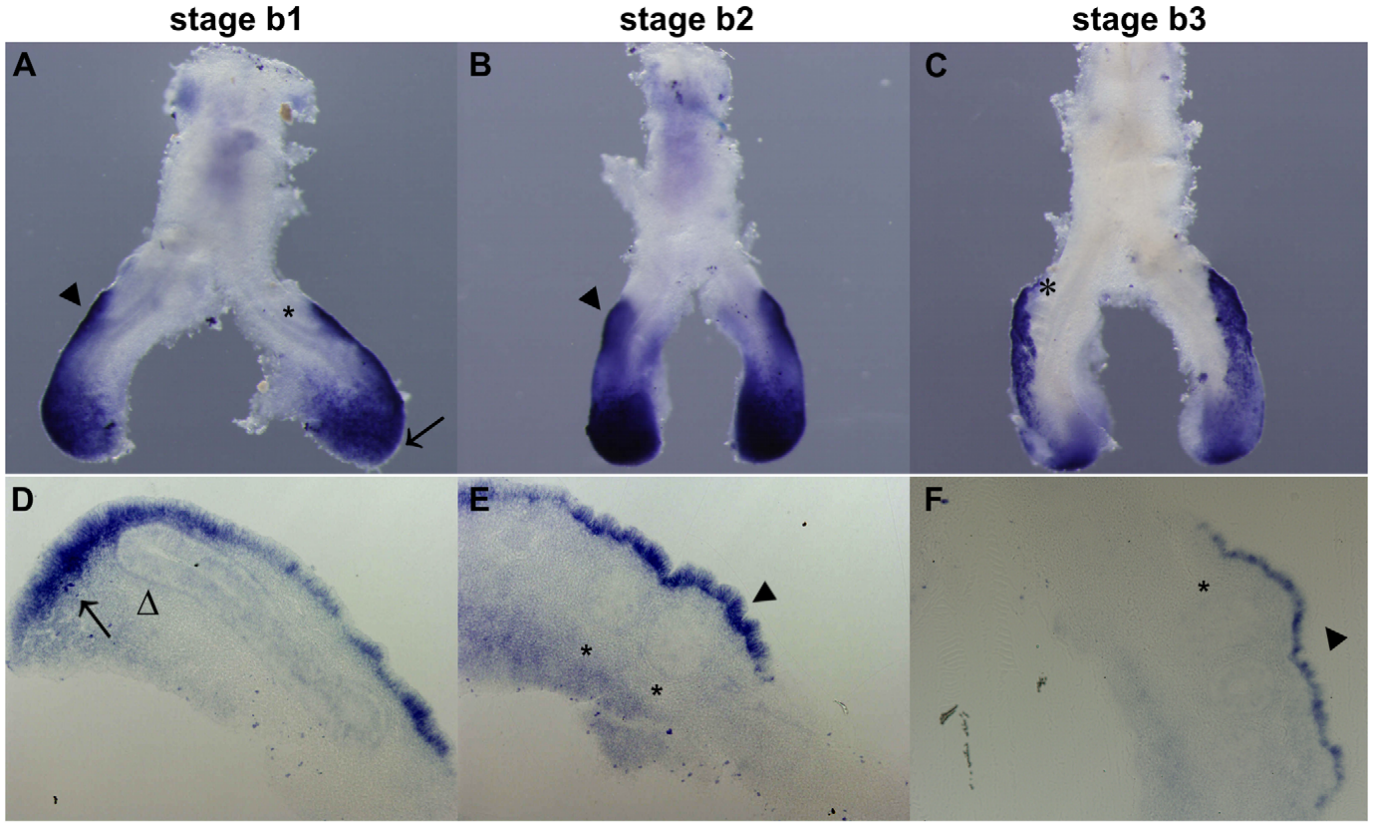
*fgf10* expression pattern in early stages of chick lung development. Representative examples of whole mount *in situ* hybridization (**A–C**) and sections of hybridized lungs (**D–F**). *fgf10* is expressed in the mesenchyme surrounding the emerging new buds (dark arrowheads) and in the distal mesenchyme surrounding the main bronchus (arrow); no epithelial expression is observed in either the main bronchus (open arrowhead) or in secondary buds (asterisks). Magnification: A - 6.3×; B/C - 5×; D/F - 10×.

We found that *fgf10* is expressed in the distal pulmonary mesenchyme surrounding the tip of the main bronchus (Figure 1A and D, arrows), and also in the dorsal mesenchyme adjacent to the emerging secondary buds, in the three stages studied (Figure 1, dark arrowheads). No epithelial expression is observed (Figure 1, open arrowhead and asterisks).

### Expression patterns of *fgfr1*, *fgfr2*, *fgfr3 and fgfr4* during chick lung development

**Figure 2:**
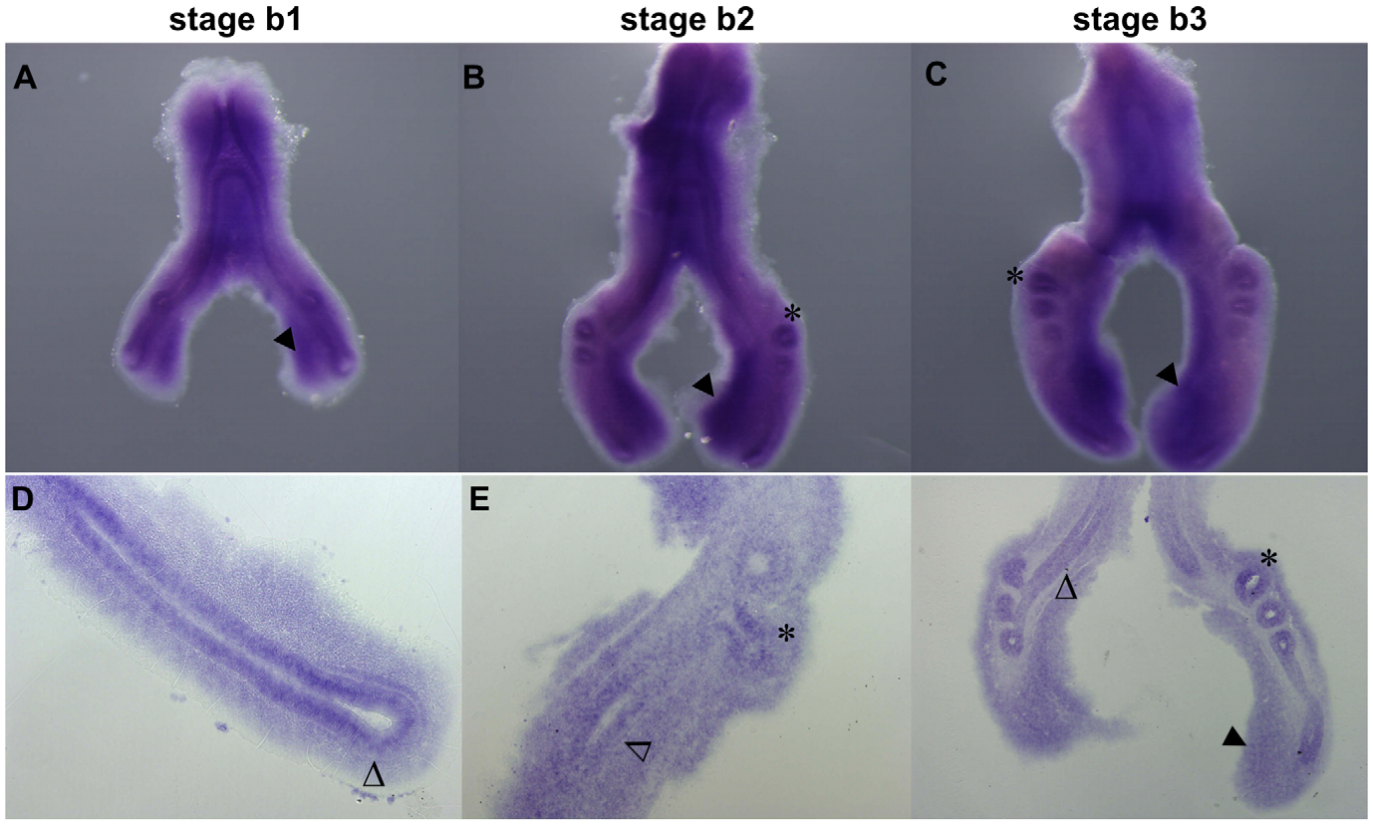
*fgfr1* expression pattern in early stages of chick lung development. Representative examples of whole mount *in situ* hybridization (**A–C**) and sections of hybridized lungs (**D–F**). *fgfr1* presents a ubiquitous expression pattern observed in the pulmonary epithelium of the main bronchus (open arrowheads) and in secondary buds (asterisks); and in distal ventral mesenchyme (dark arrowheads). Magnification: A/C - 5×; D/E - 10×; F - 4×.

*fgfr1* is expressed throughout all pulmonary epithelium and mesenchyme of the chick respiratory tract, in all stages studied (Figure 2). Histological sectioning of hybridized lungs confirmed this ubiquitous expression: *fgfr1* transcript is present in the pulmonary epithelium of the main bronchus (Figure 2D–F, open arrowhead) and of emerging secondary branches (Figure 2E and F, asterisks); moreover, it is observed over all mesenchymal compartments, mainly in the posterior ventral region (Figure 2F, dark arrowhead).

**Figure 3:**
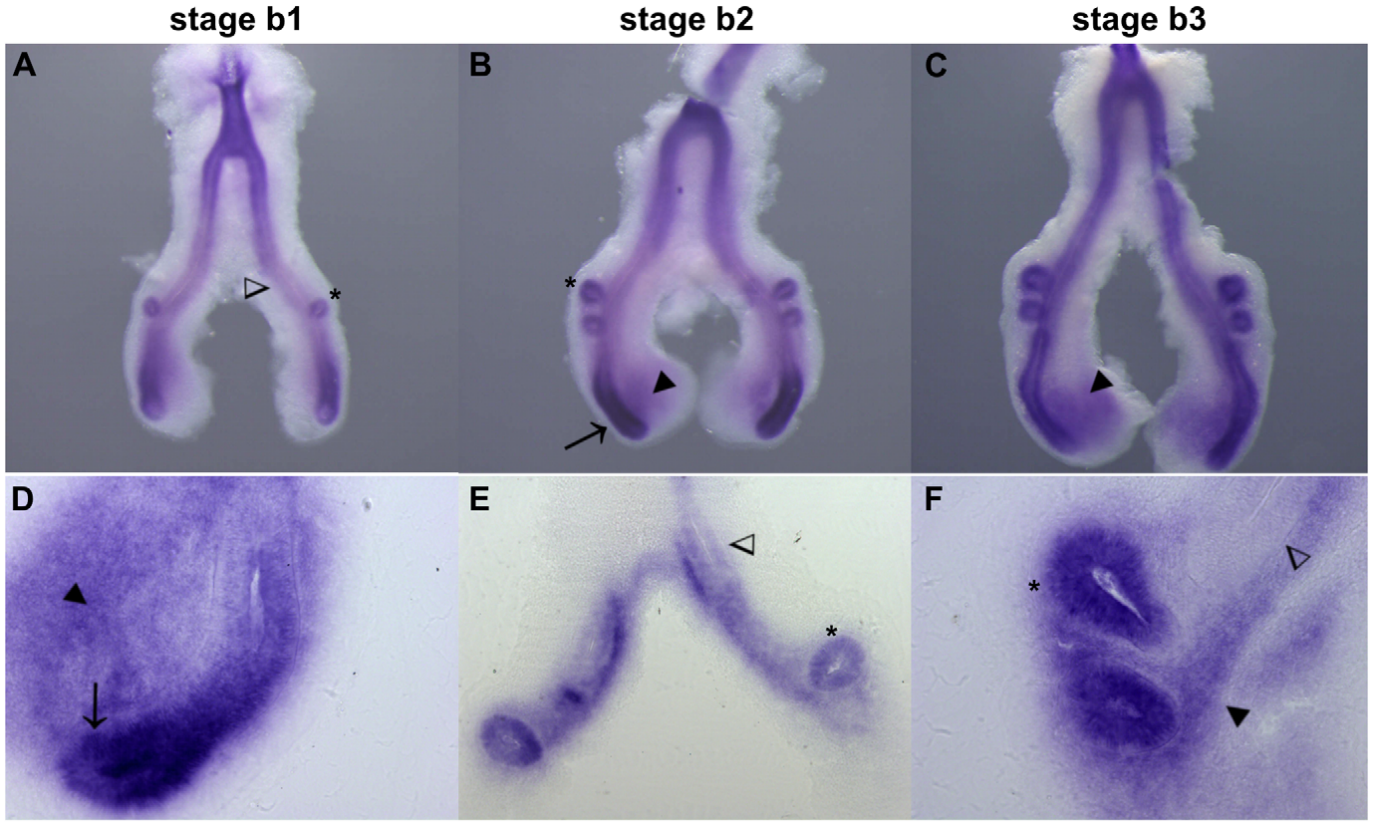
*fgfr2* expression pattern in early stages of chick lung development. Representative examples of whole mount *in situ* hybridization (**A–C**) and sections of hybridized lungs (**D–F**). *fgfr2* is present in distal epithelium of main bronchus (arrow) and secondary bronchi (asterisks); peri-epithelial mesenchyme of the main bronchus (dark arrowheads); open arrowheads, proximal epithelium. Magnification: A/C - 5×; D, F - 20×; E - 10×.

*fgfr2* is clearly expressed in the lung epithelium, mainly in distal-most region of the main bronchi (Figure 3, arrows) and in the secondary lung buds (Figure 3, asterisks). Furthermore, it is also present in distal peri-epithelial mesenchyme of the main bronchi (Figure 3, dark arrowheads). No differences were observed in *fgfr2* expression patterns in the three stages studied. In lung sections, it is possible to observe that *fgfr2* is less expressed in the most proximal epithelium (Figure 3E and F, open arrowheads) when compared with secondary bronchi epithelium (Figure 3E and F, asterisks) and distal epithelium of the main bronchus (Figure 3D, arrow). Moreover, it is expressed in the surrounding peri-epithelial mesenchyme of both main and secondary bronchi (Figure 3D and F, dark arrowheads).

**Figure 4:**
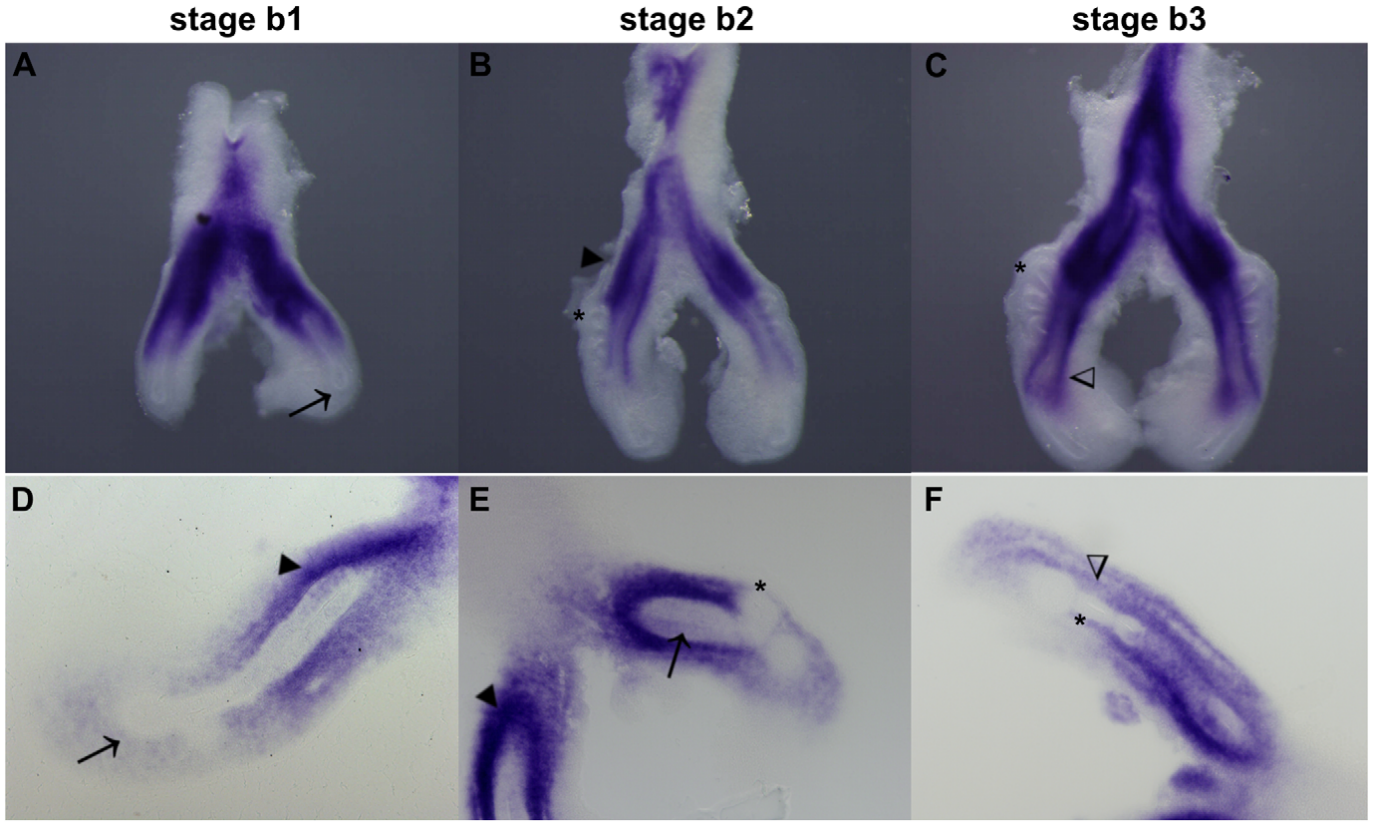
*fgfr3* expression pattern in early stages of chick lung development. Representative examples of whole mount *in situ* hybridization (**A–C**) and sections of hybridized lungs (**D–F**). *fgfr3* is absent from pulmonary epithelium (arrow, main bronchus; asterisks, secondary buds) and distal mesenchyme but expression is observed in the peri-epithelial mesenchyme (dark arrowheads, open arrowheads). Magnification: A/C - 5×; D/F - 10×.

*fgfr3* mRNA is detected in the peri-epithelial mesenchyme of the main bronchial tree, mostly in the proximal region adjacent to the emerging secondary bronchi (Figure 4, dark arrowheads). *fgfr3* transcript is absent from the pulmonary epithelium (Figure 4, arrows and asterisks), and also from the distal mesenchyme (Figure 4B). As the number of secondary bronchi increases, mesenchymal peri-epithelial *fgfr3* expression becomes progressively more distal (Figure 4, open arrowheads). Histological sectioning of hybridized lungs confirmed that *fgfr3* is not expressed in lung epithelium (Figure 4, arrows and asterisks) and is only expressed in the mesenchyme (Figure 4, dark and open arrowheads).

**Figure 5:**
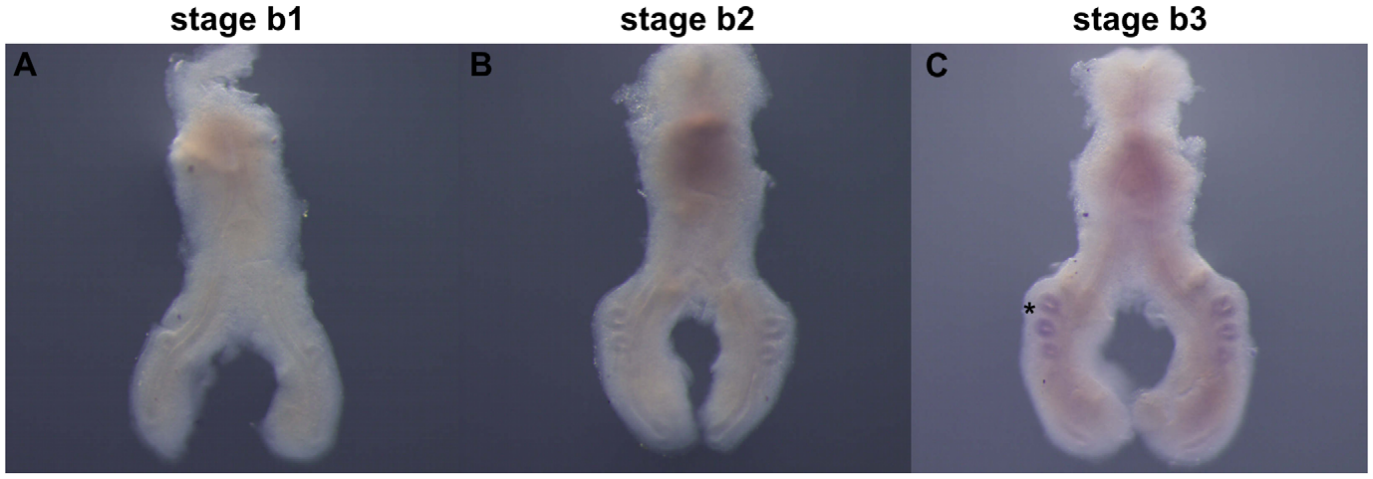
*fgfr4* expression pattern in early stages of chick lung development. Representative examples of whole mount *in situ* hybridization (**A–C**). *fgfr4* is not expressed in b1 and b2 stages. A weak expression is observed in secondary lung buds (asterisk) of b3 stage (**C**). Magnification: A/C - 5×.

*fgfr4* appears to be expressed at a much lower level in the chick respiratory tract in the stages studied. A very weak expression in the epithelium of the secondary buds in b3 stage (Figure 5C, asterisk) seems to come out, after a long developing period. Mesenchyme seems clean even after a long period of incubation with developing solution. Sectioning was not performed because the low levels of expression would be undetectable.

### Expression pattern of *spry2* during chick lung development

**Figure 6:**
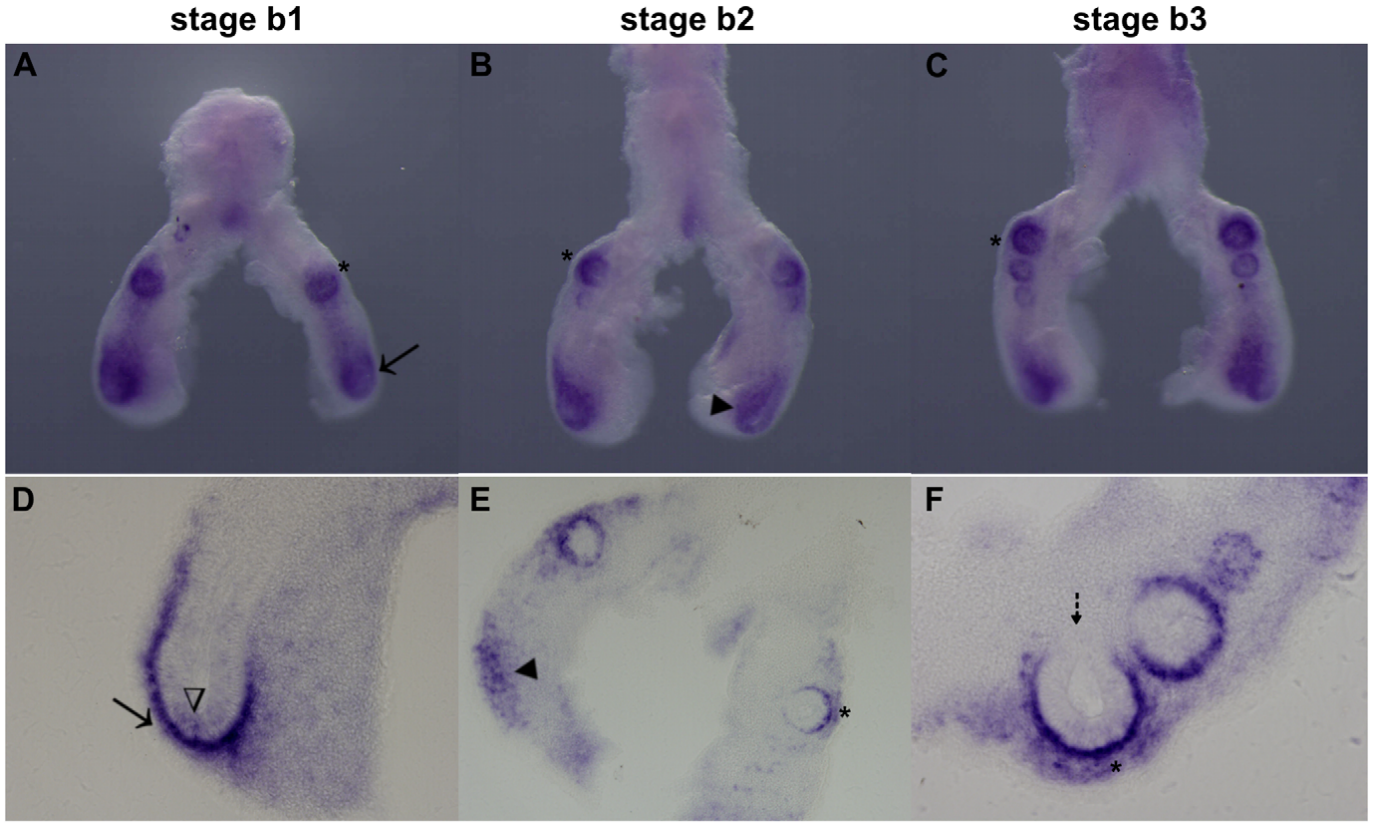
*spry2* expression pattern in early stages of chick lung development. Representative examples of whole mount *in situ* hybridization (**A–C**) and sections of hybridized lungs (**D–F**). *spry2* is present in the pulmonary epithelium (arrow, distal main bronchus; asterisks, secondary buds) and in the distal peri-epithelial mesenchyme (dark arrowheads). Open arrowheads and dashed arrow, epithelial cells. Magnification: A/C - 5×; D, F - 20×; E - 10×.

*spry2* mRNA is present in the distal epithelium of the main bronchi (Figure 6, arrows), in the epithelium of the emerging secondary buds (Figure 6, asterisks) and also in the distal peri-epithelial mesenchyme (Figure 6B and 6E, dark arrowhead). This expression pattern is constant for the three stages studied. In lung sections it is possible to observe that *spry2* is expressed not only in the distal-most epithelial cells (Figure 6D, open arrowhead), but also in the adjacent layer of mesenchyme that borders growing lung epithelium. Finally, the epithelial cells attached to the main bronchus do not express *spry2* (Figure 6F, dashed arrow).

### Inhibition of FGF signaling results in abnormal lung branching

**Figure 7:**
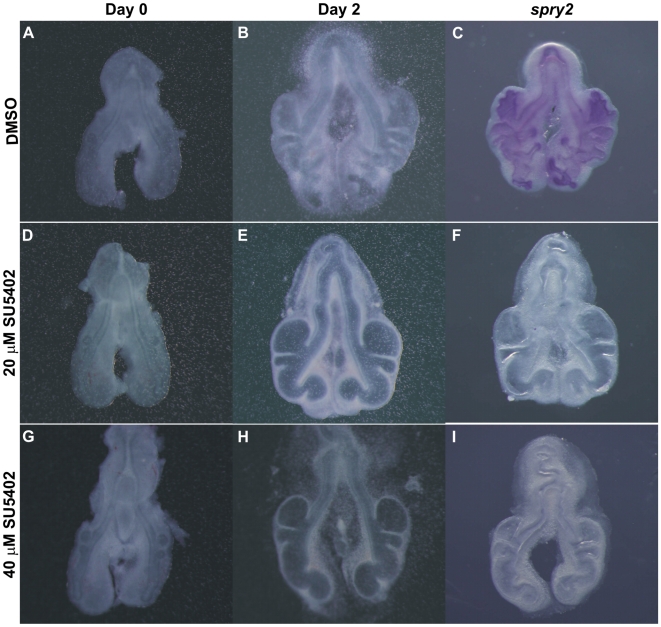
*In vitro* FGF inhibition studies in chick lung explants. Representative examples of stage b2 lung explant culture (A–B; D–E; G–H) and ISH for *spry2* (C, F, I). D0: 0 h, D2: 48 h of incubation. A–B, DMSO treated explants. D–E and G–H, SU5402 treated explants with 20 and 40 µM, respectively. All images at the same magnification: 5×.

In order to assess the role of FGF signaling pathway in early stages of chick lung development, *in vitro* inhibition studies were performed. Chick lungs from b1 to b3 stages were processed for explant culture and incubated with two different doses of a FGF receptor antagonist (SU5402), which is known to inhibit tyrosine kinase activity of all four FGFRs by interacting with the catalytic domain and thus blocking FGF signaling [19]. When compared with DMSO controls (Figure 7A–C), SU5402 treated lungs do not form new secondary branches after 48 h of incubation (Figure 7D–I). Moreover, in SU5402 treated explants, secondary branches present a cystic shape and the pulmonary mesenchyme is thinner when compared with DMSO treated explants. Cell death was assessed in these experimental conditions by TUNEL assay (Figure S1). We found that apoptosis was absent from untreated, DMSO and 20 µM treated explants and in 40 µM treated explants, only minor cellular apoptosis levels were detected in the most distal part of the lung. Hence, the described phenotypes are not due to increased cell death in lung explants.

**Figure 8:**
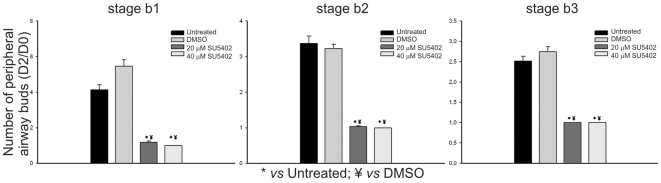
Branching analysis of stage b1, b2 and b3 lung explants treated with DMSO (n = 10), SU5402 (20 µM and 40 µM) (n = 10), or grown without supplementation (untreated, n = 10). Results are expressed as D2/D0 ratio. Data is represented as mean ± SD. p<0.05: * *vs* Untreated, ¥ *vs* DMSO.

**Figure 9:**
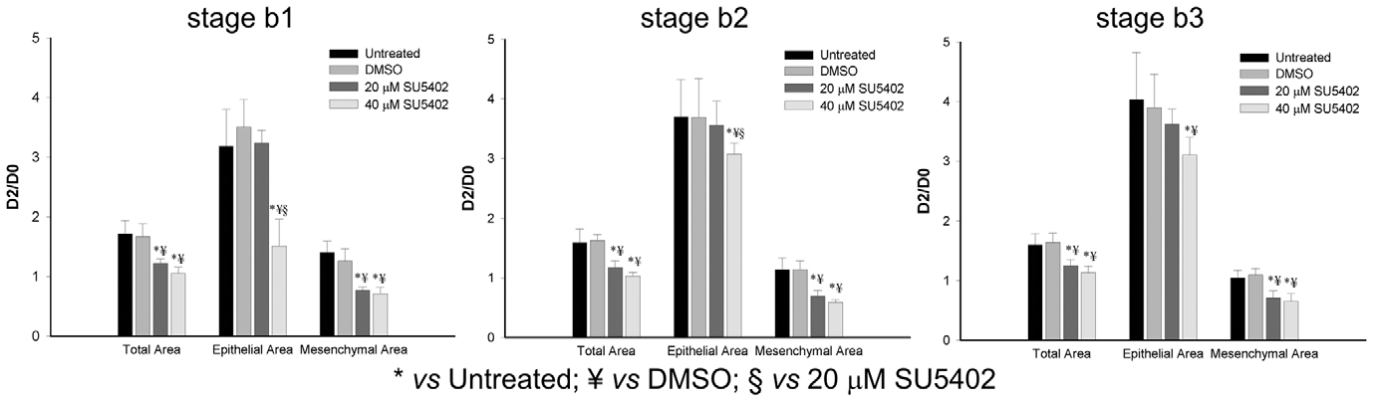
Morphometric analysis of stage b1, b2 and b3 lung explants treated with DMSO (n = 10), SU5402 (20 µM and 40 µM) (n = 10), or grown without supplementation (untreated, n = 10). Results are expressed as D2/D0 ratio. Data is represented as mean ± SD. p<0.05: * *vs* Untreated, ¥ *vs* DMSO, § *vs* 20 µM of SU5402.

To confirm FGF signaling inhibition, treated explants were hybridized with *spry2* probe, a direct readout of FGF signaling activity in the lung. As expected, SU5402 treated explants lack *spry2* expression, meaning that FGF inhibition was successful in the doses tested (Figure 7F and I), whereas in DMSO treated explants *spry2* is present (Figure 7C). The branching analysis of the fetal lung explants is summarized in Figure 8. Both doses of FGFR inhibitor induced a statistically significant difference (*p*<0.05) in the ratio of total number of peripheral airway buds (D2/D0) when compared to both untreated and DMSO treated explants, in the three stages studied. Moreover, no statistical differences were found between groups treated with 20 and 40 µM of SU5402 for all stages studied. Morphometric analysis was performed by assessing total, epithelial and mesenchymal lung explant area (Figure 9). Both doses of FGFR inhibitor induced a statistically significant reduction (*p*<0.05) in total and mesenchymal area ratio (D2/D0) when compared to both untreated and DMSO treated explants, in the three stages studied; in these conditions, no statistical differences were found between groups treated with 20 or 40 µM of SU5402 in all stages studied. On the other hand, regarding epithelial area ratio, only the higher dose of inhibitor induced a statistically significant decrease (*p*<0.05) when compared to both untreated and DMSO treated explants, in the three stages studied.

## Discussion

FGF signaling pathway is crucial for proper embryonic development and it is known to play an important role in several stages of lung morphogenesis (reviewed in [8]). Lung development is directed by intrinsic epithelial-mesenchymal interactions that regulate cell proliferation, fate, migration and differentiation, leading to branching morphogenesis and ultimately to proper lung formation. These signaling events are coordinated by bone morphogenetic proteins (BMP), fibroblast growth factors (FGF), sonic hedgehog (SHH) and Wnt signaling pathways, insoluble extracellular matrix proteins and their receptors, as well as various transcription factors [1]. In this study we analyze for the first time, the expression pattern of some members of the FGF signaling pathway in early stages of chick lung development, such as *fgf10*, *fgfr1-4* and *spry2*. Moreover, FGF inhibition studies were also performed in order to assess FGF functional role in branching morphogenesis.

Although the anatomy of the avian lung differs from that of the mammalian lung, both develop similarly and have anatomical functional equivalents. Mammalian lung is asymmetrical and branching occurs by reiterative dichotomous splitting of the airway epithelium into surrounding mesenchyme, generating a tree-like structure that will give rise to the airways and alveoli [20]. On the other hand, chick lung is symmetrical and two developmental processes take place so the lung forms properly: secondary bronchi sprout along the length of the primary bronchus and then tertiary bronchi anastomose and interconnect with the secondary bronchi. Ventrally, avian lung branches end in air sacs [21]. In both cases, however, the airways develop by a succession of outgrowths and extensions into the surrounding mesenchyme, which could be governed by similar molecular mechanisms.

### Expression pattern of FGF signaling members

We found that *fgf10* is expressed in the distal mesenchyme of the main bronchus and in the dorsal mesenchyme that surrounds the emerging secondary buds (Figure 1). This expression pattern is consistent with what has been previously described in the mouse model: *fgf10* is expressed in the distal mesenchyme at sites where prospective epithelial buds will appear governing the directional outgrowth of lung buds during branching morphogenesis [10]. These data suggest that *fgf10* may also be important for chick airway development, functioning as a proliferative factor that stimulates distal epithelial growth.

*fgfr1* shows an ubiquitous expression in the epithelial and mesenchymal compartments that is consistent with that described for rat lung development (Figure 2) [22], [23]. This expression pattern suggests that FGFR1 could be responsible for capturing proliferative factors, such as FGF9 that acts as a trophic factor for distal mesenchyme, and FGF10 which is important for epithelial proliferation, although through different isoforms, FGFR1c and 1b respectively [1].

*fgfr2* is expressed throughout the epithelial compartment, particularly in the most distal region of the main bronchus and also in the growing secondary branches (Figure 3). This expression pattern matches the one described for the mouse lung [10] and suggests that, also in the chick model, FGFR2 might be activated by FGF10 during the embryonic phase of development [11]. FGFR2 also binds to other ligands (FGF1, FGF3, FGF7), which are primarily expressed in mesenchymal cells. Some mesenchymal expression is also observed in the most distal peri-epithelial region that might be activated by FGF1 [24], probably through the FGFR2c isoform [25].

*fgfr3* is present in the peri-epithelial mesenchyme of the bronchial airway, mainly anteriorly to the emerging secondary buds (Figure 4). Considering the fact that in later stages its expression is progressively more distal, this might mean that it is responsible for the uptake of differentiating growth factors with an inhibitory growth action. This inhibitory function has already been described in mouse chondrocyte proliferation and differentiation [26].

**Figure 10:**
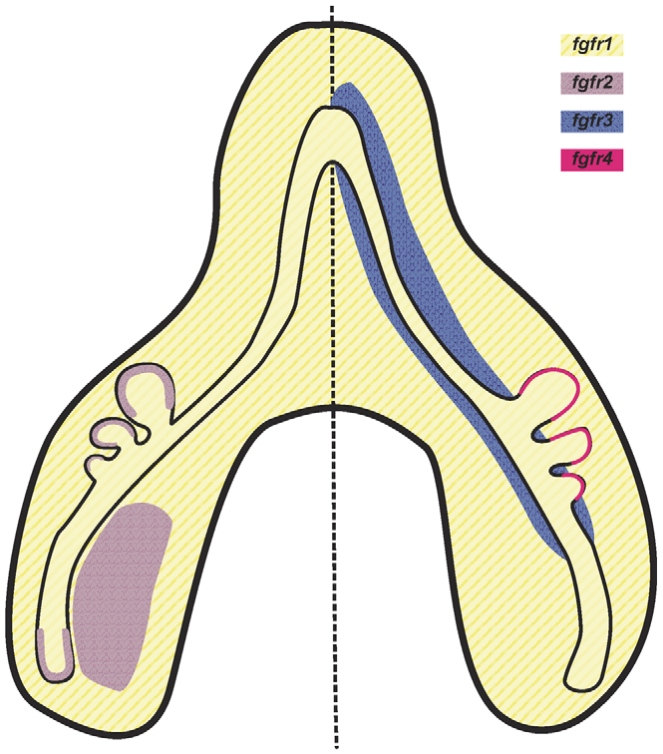
Schematic diagram of FGF receptor expression pattern in a b3 stage lung. For simplicity, image was divided in half in order to avoid color overlay. Yellow: *fgfr1* expression; purple: *fgfr2* expression; blue: *fgfr3* expression; pink: *fgfr4* expression.

Regarding *fgfr4* expression, only a very faint expression was observed in the secondary buds in the later stages (Figure 5). These results are in accordance with the fact that this receptor binds to several FGFs which have not been implicated in lung development. Moreover, the presence of *fgfr4* could only be detected in pre-natal and early post-natal rat lungs [23]. It is also known that *fgfr3/fgfr4* knockout present late defects in pulmonary development while single *fgfr3* and *fgfr4* knockouts do not show any lung phenotype [27]. Considering the results obtained and the fact that in the adult lung *fgfr4* expression is absent, maybe this receptor has a role only in the late stages. A summary diagram of the four FGF receptor expression patterns is represented in Figure 10.

*spry2* is localized to the distal tips of the main bronchus and secondary branches (Figure 6). This expression pattern is in accordance with what has been described in the literature for rodent models where *spry2* is expressed in the distal tip, as the bud elongates toward a mesenchymal source of FGF10 [28]. Therefore it seems that, also in the chick lung, *spry2* is expressed in intimate association with a FGF10 signaling center. In addition to the distal epithelial expression, similar to what occurs in rodent models, *spry2* is also significantly expressed in mesenchyme that surrounds growing epithelial branches. The preservation of a distal expression pattern, however, sustains the hypothesis that the FGF10-Spry2 system is also regulating lung branching morphogenesis in the chick lung.

### FGF signaling inhibition

*In vitro* inhibition of FGF signaling by SU5402, a known inhibitor of all FGF receptors, induced an abnormal lung growth with a cystic appearance of secondary bronchi and a disruption of the mesenchymal tissue (Figure 7). As stated previously, FGF signaling plays an important role in lung branching morphogenesis but so far these effects have not been described in the chick embryonic lung. To demonstrate that this morphological alteration was due to FGFR inhibition, lung explants were probed for *spry2* expression. As expected, SU5402 treated explants lack *spry2* mRNA, supporting that FGF signaling is down-regulated, while in DMSO treated explants (Figure 7C) and lung explants with no supplementation (data not shown), *spry2* is normally expressed. Also, explants treated with SU5402 exhibit a dysplastic structure without an increase in the number of secondary bronchi after 48 h of culture (expressed as D2/D0 ratio, Figure 8). However, we cannot rule out the possibility that other signaling pathways may be inhibited in these culture conditions, also contributing for the observed explant morphology. Vascular endothelial growth factor receptor (VEGFR) is known to be required for pulmonary vascular development and epithelial branching [29] and is inhibited by SU5402 [30]. Also, when this signaling pathway is blocked in fetal rat lung explants by a specific VEGFR inhibitor, a slight increase in distal air space size is observed [31], although not to the extent observed in the case of chick lung explants (our results).

Morphometric analysis of lung explants (Figure 9) clearly shows that both doses of FGFR inhibitor induced a significant reduction of the mesenchymal area, in the three stages studied. On the other hand, the epithelial compartment seems to be less susceptible to SU5402 inhibition since only the higher dose induced a statistical difference. Considering the role of FGFs as proliferation factors, it seems as if the mesenchyme is more sensitive to these extracellular proteins than the epithelium.

Regarding the cystic shape of lung buds, theoretically there are two possible explanations for this phenotype. It has been suggested that either an increase in FGF diffusion rate or a saturating amount of FGF signal should result in cyst formation [32]. In fact, transiently induced FGF overexpression during fetal rat lung development leads to the formation of cystic lung lesions [33]. On the other hand, increased FGF diffusion accounts for the formation of air sacs in the ventral chick lung [18]. Apparently, none of these two situations match the experimental conditions described in this work, however, an increase in FGF diffusion due to the disrupted mesenchyme structure could contribute to the cystic phenotype observed.

In conclusion, our work demonstrates the importance of FGFRs in the epithelial-mesenchymal interactions that determine epithelial branching and mesenchyme growth and consolidate our understanding of the role of FGF signaling pathway in early chick lung development.

## Materials and Methods

### Eggs and embryos

Fertilized chick (*Gallus gallus*) eggs, obtained from commercial sources, were incubated for 4–6 days in a 49% humidified atmosphere at 37°C. Embryonic chick lungs were carefully dissected under a dissection microscope (Olympus SZX16, Japan) and were then classified as stage b1, b2 or b3, taking into account the number of secondary buds formed: 1, 2 or 3, respectively.

### *In situ* hybridization

Lungs were fixed overnight at 4°C in a solution of 4% formaldehyde with 2 mM EGTA in PBS at pH 7.5, rinsed in PBT (PBS, 0.1% Tween 20), dehydrated in methanol and stored at −20°C. Whole mount *in situ* hybridization was performed as previously described by Henrique et al. [34]. Lungs were then photographed in PBT/0.1% azide with an Olympus U-LH100HG camera coupled to Olympus SZX16 stereo microscope. Hybridized chick lungs were fixed in paraformaldehyde 4%, embedded in 2-hydroxyethyl methacrylate (Heraeus Kulzer, Germany) and processed for sectioning at 25 µm using a rotary microtome (Leica RM 2155, Germany). Lung sections were photographed with an Olympus DP70 camera coupled to an Olympus BX61 microscope.

Non isoform-specific FgfR1-4 probes were kindly provided by Dr. Guojun Sheng [35]. Fgf10 probe was kindly provided by Dr. Ana Certal (IGC, Portugal) and Spry2 probe by Dr. Delphine Duprez [36]. Digoxigenin (DIG)-labeled probes were synthesized using linearized plasmids and DIG RNA Labeling Mix (Roche Applied Sciences, Germany) according to the manufacturer's instructions.

### Lung Explant Culture

After dissection in DPBS (Lonza, Switzerland), lungs were transferred to Nucleopore polycarbonate membranes with an 8 µm pore size (Whatman, USA) and incubated in a 24-well culture plates (Orange Scientific, Belgium). The membranes were presoaked in 400 µL of Medium 199 (Sigma, USA) for 1 h before the explants were placed on them. Floating cultures of the explants were incubated in 200 µL Medium 199 supplemented with 5% heat inactivated fetal calf serum (Invitrogen, USA), 10% chicken serum (Invitrogen), 1% L-glutamine (Invitrogen), 1% penicillin 5000 IU/mL, streptomycin 5000 IU/mL (Invitrogen) and 0.25 mg/mL ascorbic acid (Sigma). Chick lung explants were incubated in a 5% CO_2_ incubator at 37°C for 48 h and the medium replaced at 24 h. The branching morphogenesis was monitored daily by photographing the explants. At day 0 (D0: 0 h) and day 2 (D2: 48 h) of culture, the total number of peripheral airway buds (branching) was determined. For the morphometric analysis, the total and epithelial areas were assessed at D0 and D2 using AxionVision Rel. 4.3 (Carl Zeiss GmbH, Germany). Epithelial area was defined by the internal perimeter of the lung and total cross-sectional area was defined by the outer perimeter; mesenchymal area was calculated as the difference between total and epithelial area. Branching and morphometric results were expressed as D2/D0 ratio. All quantitative data are presented as mean ± SD. Statistical analysis was performed, using SigmaStat 3.5 (Systat Software Inc., USA), by one-way ANOVA On Ranks and the Dunn test (branching) and Turkey test (morphometric) were carried out for post-test analysis. Statistical significance was set at p<0.05.

In order to inhibit the FGF signaling pathway, lung explants (stages b1 to b3) were cultured with SU5402 (Calbiochem, UK), an FGF receptor antagonist; SU5402 dissolved in DMSO was added to the medium to achieve a final concentration of 20 and 40 µM (n = 10, per stage) and 0.1% DMSO. Control explants consisted of medium containing DMSO at a final concentration of 1 µL/mL (n = 10, per stage). After culture, lung explants were fixed overnight at 4°C and processed for ISH.

## Supporting Information

Figure S1 **TUNEL assay in chick lung explants.** Apoptosis was analyzed using the Cell Death Detection Kit (Roche Applied Sciences) in all experimental groups. Briefly, explants were fixed overnight in 4% paraformaldehyde (PFA) in PBS, permeabilized with PBS/0.5% Triton X-100/0.1% sodium citrate for 4 h at room temperature, and washed in PBS. Positive control explants were incubated with DNase at 37°C for 1 h. Explants were incubated for 4 h, at 37°C with the TUNEL solution mix and washed at least three times in PBS before visualization. Representative examples of positive control (A), DMSO (B), 20 and 40 µM SU5402 (C and D, respectively) treated stage b3 explants. Apoptosis is absent from untreated (data not shown), DMSO and 20 µM SU5402 treated explants (B and C, respectively). In 40 µM treated explants (D) only minor cellular apoptosis levels were detected in the most distal part of the lung. White arrows point to specific TUNEL staining.
(TIF)
