# Supplementary figures and images for: FGF Signaling Pathway in the Developing Chick Lung: Expression and Inhibition Studies
Source: PLoS One. 2011 Mar 11;6(3):e17660. doi: 10.1371/journal.pone.0017660 (PMC3055888; doi:10.1371/journal.pone.0017660)

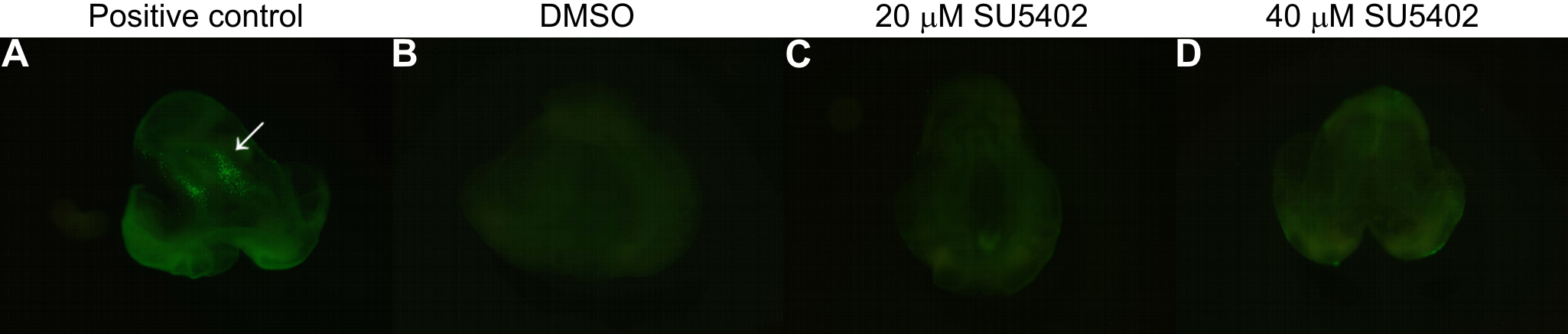

Supplement: Figure S1 — TUNEL assay in chick lung explants. Apoptosis was analyzed using the Cell Death Detection Kit (Roche Applied Sciences) in all experimental groups. Briefly, explants were fixed overnight in 4% paraformaldehyde (PFA) in PBS, permeabilized with PBS/0.5% Triton X-100/0.1% sodium citrate for 4 h at room temperature, and washed in PBS. Positive control explants were incubated with DNase at 37°C for 1 h. Explants were incubated for 4 h, at 37°C with the TUNEL solution mix and washed at least three times in PBS before visualization. Representative examples of positive control (A), DMSO (B), 20 and 40 µM SU5402 (C and D, respectively) treated stage b3 explants. Apoptosis is absent from untreated (data not shown), DMSO and 20 µM SU5402 treated explants (B and C, respectively). In 40 µM treated explants (D) only minor cellular apoptosis levels were detected in the most distal part of the lung. White arrows point to specific TUNEL staining. (TIF) [file pone.0017660.s001.tif]
